# Doxycycline-encapsulated solid lipid nanoparticles as promising tool against *Brucella melitensis* enclosed in macrophage: a pharmacodynamics study on J774A.1 cell line

**DOI:** 10.1186/s13756-019-0504-8

**Published:** 2019-04-03

**Authors:** Seyed Mostafa Hosseini, Roghayyeh Abbasalipourkabir, Farid Azizi Jalilian, Sara Soleimani Asl, Abbas Farmany, Ghodratollah Roshanaei, Mohammad Reza Arabestani

**Affiliations:** 1 Department of Microbiology, Faculty of Medicine, Hamadan University of Medical Sciences, Shahid fahmideh street, Park Mardome, Hamadan, IR Iran; 2 Department of Clinical Biochemistry, Faculty of Medicine, Hamadan University of Medical Sciences, Shahid fahmideh street, Park Mardome, Hamadan, IR Iran; 3 Department of Virology, Faculty of Medicine, Hamadan University of Medical Sciences, Shahid fahmideh street, Park Mardome, Hamadan, IR Iran; 4 Department of Anatomical Sciences, Faculty of Medicine, Hamadan University of Medical Sciences, Shahid fahmideh street, Park Mardome, Hamadan, IR Iran; 5 Dental Research Center, School of Dentistry,, Hamadan University of Medical Sciences, Shahid fahmideh street, Park Mardome, Hamadan, IR Iran; 6 Department of Biostatistics, School of Health, Hamadan University of Medical Sciences, Shahid fahmideh street, Park Mardome, Hamadan, IR Iran; 7 Brucellosis Research Center, Hamadan University of Medical Sciences, Shahid fahmideh street, Park Mardome, Hamadan, IR Iran

**Keywords:** *Brucella melitensis*, Doxycycline, Solid lipid nanoparticle, Relapse

## Abstract

**Background:**

Brucellosis is a zoonotic disease caused by *Brucella* species. It has been estimated that more than 500,000 new cases of Brucellosis occur annually all around the world. Relapse of the disease is one of the most important challenges. The most important reason for the relapse of brucellosis is the survival of the bacteria inside the macrophages, which makes them safe from the immune system and disrupts drug delivery mechanism.

**Objectives:**

The present study was performed to assess the effects of Doxycycline-loaded Solid Lipid Nanoparticles (DOX-SLN) on the *Brucella melitensis* inside macrophages.

**Methods:**

DOX-SLN was prepared using double emulsion method. The technological characterization of DOX-SLN, including particle size, zeta potential, polydispersity index (PDI), drug loading and encapsulation efficiency were used. Fourier-transform infrared spectroscopy (FTIR) and Differential scanning calorimetry (DSC) were used to assess the interactions between Nanoparticles (NPs) components and crystalline form of doxycycline. Moreover, the effect of DOX-SLN on the bacteria were compared with that of the doxycycline using various methods, including well diffusion, Minimum Inhibitory Concentration (MIC), and investigation of their effects on murine macrophage-like cells cell line J774A.1.

**Results:**

The means of particle size, zeta potential, PDI, drug loading and encapsulation efficiency were 299 ± 34 nm, − 28.7 ± 3.2 mV, 0.29 ± 0.027, 11.2 ± 1.3%, and 94.9 ± 3.2%, respectively. The morphology of NPs were spherical with a smooth surface. No chemical reaction was occurred between the components. Doxycycline was located within NP matrix in its molecular form. The DOX-SLN significantly decreased the microbial loading within macrophages (3.5 Log) in comparison with the free doxycycline.

**Conclusions:**

Since the DOX-SLN showed better effects on *B. melitensis* enclosed in macrophages than the free doxycycline, it is recommended to use it for treating brucellosis and preventing relapse.

## Background

Brucellosis is a zoonotic disease caused by different *Brucella* strains following direct or indirect contact of human with infected animals or dairy products [1]. The most common cause of the disease is *Brucella melitensis*, which is endemic in Middle East countries, including Iran. *Brucella* is an intracellular bacterium [2]. The phagocytosis activity of polymorphonuclear and monomorphonuclear cells such as macrophages plays an important role in the disease treatment [3]. Upon entering the body, about 90% of the bacteria were killed by neutrophils, monocytes, and macrophages. However, some of them survive inside macrophages and find a place to proliferation within the cell [1, 4].

The World Health Organization (WHO) suggests the combination of gentamicin and doxycycline for brucellosis treatment [5]. The combination of doxycycline and streptomycin has also been suggested to prevent the disease relapse. The combination of doxycycline and rifampin for 45 days is the best known treatment for Brucellosis because of having no side effects and its acceptability by patients. However, this treatment has been less effective with about 15% failure in different types of the disease [6].

Despite the significant number of new antibiotics, treatment of intracellular pathogens is still a major challenge [7]. To date, no antibiotic therapy has been reported to eradicate *Brucella* intracellular infections. The main challenge in intracellular chemotherapy is the design and development of a carrier system for antibiotics that can be effectively endocytosed by phagocytic cells and release drugs. Targeted drug delivery system can be considered as an appropriate tool to overcome such problems [8].

One of the most promising strategies to prevent the disease recurrence is using the antibiotic-loaded nanocarriers. Nowadays, with the production of pharmaceutical NPs, unique characteristics can be achieved, which will result in increased performance of drugs and diversity of their forms.

These carrier systems have to be non-toxic and have sufficient capacity to take the drug. In addition, they should have the possibility of controlling drug release. Solid lipid nanoparticles (SLNs) as a carrier system have been investigated for many applications. These drug delivery systems provide controlled release of medicines that they carry and increase the chemical stability of them [9, 10].

In spite of negligible limitations, the most important properties of SLNs are protecting drugs from chemical and enzymatic decompositions, physical stability, hydrophilic and hydrophobic drug loading capacity, easy production, no need for organic solvents in synthesis, ability to simultaneous carry two active agents, increase drug efficiency, ease of sterilization, small diameter, administration via different routes, biodegradability and biotic stability, increase bioavailability of drug, effective pass from biological barriers, reduce dose and frequency of drug administration, fast identification by phagocytic system, controlled release of drug, specific targeting, slow release of drug in a long-term period, protect drugs against pharmacologic and toxicological effects, prevent early breakdown of drug molecules, prevent immune response, increase drug retention in tissue, minimize resistance to drug, improve therapeutic index of drug, minimize drug toxicity, and improve treatment efficiency.

Despite its partial limitations, the followings can be mentioned as the most significant features of SLNs: protecting drugs from chemical and enzymatic decompositions as well as pharmacologic and toxicological effects, physical and biotical stability, hydrophilic and hydrophobic drug loading capacity, easy production, no need for organic solvents in synthesis, ability to carry two active agents simultaneously, increase drug efficiency, easy sterilization, small diameter, administration via different routes, biodegradability trait, increase bioavailability of drug, effective pass from biological barriers, reduce dose and frequency of drug administration, fast identification by phagocytic system, controlled release of drug, specific targeting, slow release of drug in a long-term, prevent early breakdown of drug molecules, prevent immune response, increase drug retention in tissue, minimize resistance to drug, improve therapeutic index of drug, minimize drug toxicity and improve treatment efficiency [11, 12].

Given the above, the present study was carried out to develop a new therapeutic mechanism for the treatment of brucellosis in order to enhance efficacy of antibiotics. The therapeutic efficacy of doxycycline-loaded SLNs in chronic infections of *B. melitensis* was assessed in cellular models and experimental conditions.

## Methods

### Materials

Hydrogenated palm oil, (Softisan 154 or S154) a gift from Condea (Witten, Germany), and stearic acid (SA, Sigma Aldrich, USA) were the lipids used in this study. Water soluble surfactants used were polioxiethylene-20-sorbitan monooleate (Tween 80- Sigma Aldrich, USA), Poloxamer407 (Pluronic F127, Sigma Aldrich, USA). Furthermore, lipo-soluble surfactants used were Sorbitanmonooleate (Span 80, Sigma Aldrich, USA), and soy lecithin (Sigma-Aldrich). Doxycycline hyclate (Sigma-Aldrich). Water used in our study was distilled twice and purified by an appropriate filter.

### Preparation of NPs

Solid-lipid NPs (SLNs) were synthesized using the above mentioned chemicals and double emulsion/melt dispersion technique based on the study conducted by Luana Becker Peres [13]. The first emulsion contained palm oil, Poloxumer, distilled water, and doxycycline antibiotic. In brief, firstly, 0.6 g of palm oil was warmed to 60 °C (the boiling point) in Ben Murray. Then 60 mg Poloxamer and 30 mg doxycycline were added to the melted palm oil and mixed on the magnetic stirring (60 °C and 150 rpm) for 5 min. In the following step, 0.5 ml hot distilled water was added to the mixture. Mixture was sonicated using sonicator (skymen, China) during 60 s at 45% amplitude (20 W) to obtain the first emulsion. (W1/O). In the next step, 30 mg tween-80 was added to the prepared first emulsion and made homogenous using ultrasonic device (Bandelin Sonopuls, Berlin, Germany) at 45% amplitude (20w) at a regular pulse rhythm (10s on and 5 s off) for 60s to the second emulsion (W1/O/W2) be provided. The prepared second emulsion was gently added to 30 ml of cold distilled water (4 °C) under the condition of magnetic starring (5 min) to stabilize SLN.

Materials required for each formulation are presented in Table 1. Finally, doxycycline-loaded solid lipid nanoparticles (DOX-SLN) was separated using the high speed centrifuge (37,000 g for 15 min) and washed using sterilized distilled water three times. Almost 500 mg of each formulation was added to 1 ml glycerin. The prepared samples were lyophilized at − 80 °C using a vacuum pump (Christ, china) with a condenser flow. For being usable in biologic studies on bacteria and cell line, lyophilized NPs transformed in solution and sterilized using 450 nm filters [14].

**Table 1:** Materials and technological parameters of Various formulation

	sample	Lipid (mg)	Lipid surfactant (ML, Mg)	Water surfactant (ML, Mg)	Water added (ML)	PS (nm)	PDI	Zeta potential (mV)	EE (%)	DL (%)
Before lyophilization	SLN1 without doxycycline	Palm oil (600)	Lecithin (60)	Tween 80 (6)	50	251 ± 15	0.277 ± 0.021	−31.6 ± 1.4	95.4 ± 3.5	11.3 ± 1.1
	SLN 1 with doxycycline	Palm oil (600)	Lecithin (60)	Tween 80 (6)	50	265 ± 23	0.284 ± 0.012	−29.3 ± 2.1		
	SLN 2 without doxycycline	Stearic acid (600)	Span 80 (6)	Poloxamer407 (60)	100	456 ± 21	0.429 ± 0.032	−11.6 ± 4.4	93.7 ± 5.3	8.9 ± 2.3
	SLN 2 with doxycycline	Stearic acid (600)	Span 80 (6)	Poloxamer407 (60)	100	465 ± 23	0.344 ± 0.012	−14.3 ± 3.5		
	SLN 3 without doxycycline	Palm oil (1200)	Span 80 (6)	Tween 80 (3)	100	358 ± 39	0.536 ± 0.019	−9.6 ± 1.7	94.1 ± 7.1	9.2 ± 3.8
	SLN 3 with doxycycline	Palm oil (1200)	Span 80 (6)	Tween 80 (3)	100	375 ± 42	0.591 ± 0.010	−11.3 ± 2.6		
	SLN 4 without doxycycline	Stearic acid (1200)	Lecithin (60)	Tween 80 (3)	50	491 ± 25	0.447 ± 0.033	−18.6 ± 1.4	97.4 ± 4.5	10.7 ± 1.4
	SLN 4 with doxycycline	Stearic acid (1200)	Lecithin (60)	Tween 80 (3)	50	415 ± 59	0.494 ± 0.093	−19.3 ± 1.2		
After lyophilization	SLN 1 without doxycycline	Palm oil (600)	Lecithin (60)	Tween 80 (6)	50	294 ± 27	0.281 ± 0.044	−30.7 ± 2.04	94.9 ± 3.2	11.2 ± 1.3
	SLN 1 with doxycycline	Palm oil (600)	Lecithin (60)	Tween 80 (6)	50	299 ± 34	0.290 ± 0.027	−28.7 ± 3.2		
	SLN 2 without doxycycline	Stearic acid (600)	Span 80 (6)	Poloxamer407 (60)	100	491 ± 33	0.499 ± 0.042	−12.6 ± 3.5	93.9 ± 4.7	8.2 ± 1.9
	SLN 2 with doxycycline	Stearic acid (600)	Span 80 (6)	Poloxamer407 (60)	100	471 ± 34	0.412 ± 0.012	−14.3 ± 3.5		
	SLN 3 without doxycycline	Palm oil (1200)	Span 80 (6)	Tween 80 (3)	100	378 ± 28	0.591 ± 0.053	−17.6 ± 2.1	91.3 ± 2.7	8.8 ± 3.1
	SLN 3 with doxycycline	Palm oil (1200)	Span 80 (6)	Tween 80 (3)	100	399 ± 39	0.612 ± 0.125	−15.3 ± 1.7		
	SLN 4 without doxycycline	stearic acid (1200)	Lecithin (60)	Tween 80 (3)	50	511 ± 45	0.493 ± 0.325	−17.8 ± 1.7	96.1 ± 3.9	9.9 ± 1.7
	SLN 4 with doxycycline	stearic acid (1200)	Lecithin (60)	Tween 80 (3)	50	575 ± 39	0.524 ± 0.987	−16.3 ± 2.2		

*PS* particle size, *PDI* polydispersity index, *EE* entrapment efficiency, *DL* drug loading

### Characteristics of NPs (particle size, polydispersity index, and zeta potential)

Particle size, Polydispersity Index, and Zeta Potential were determined by Dynamic Light Scattering (DLS) technique performed by Zetasizer Nano ZS 3600 (Malvern Instruments, Worcestershire, United Kingdom) device. The mentioned factors were measured twice; firstly, immediately after preparation, and secondly, after lyophilization [13].

### Morphology

Field emission scanning electronic microscope (FE-SEM) was used for assessing the morphological characteristics of NPs. To do so, 10 mg lyophilized DOX-SLN was added to 1 ml distilled water and then 2 μl of this suspension was placed on a glass surface. Since the suspension was dried, it was covered by a thin gold layer to avoid electrostatic charging during examination and analyzed using FE-SEM (TSCAN, Czech Republic) [15].

### Investigation of capsulated and loaded drug

For determining how much the doxycycline have been loaded and encapsulated in the synthesized NPs, an indirect method, i.e. spectrophotometer, and a direct one, i.e. high-performance liquid chromatography (HPLC), were employed in accordance with guidelines recommended in literature. In this step, 10 mg of prepared NPs was added to 10 ml distilled water and vortexed to become homogenous. Then, the solution was centrifuged at 4 °C for 20 min at 1500 rpm. The supernatant was investigated using spectrophotometer (2100 UV, USA) at a wavelength of 270 nm. The drug concentration was determined using the standard curve which have been previously depicted based on various concentrations of the drug [14, 15]. The amount of loaded and encapsulated drug was obtained using the following equations:

$$ \mathrm{Entrapment}\ \mathrm{Efficiency}\ \mathrm{EE}\left(\%\right)=\frac{\mathrm{initial}\ \mathrm{drug}\ \mathrm{amount}-\mathrm{free}\ \mathrm{drug}\ \mathrm{amount}\ }{\mathrm{initial}\ \mathrm{drug}\ \mathrm{amount}}\times 100 $$

$$ \mathrm{Drug}\ \mathrm{Loading}\ \mathrm{DL}\left(\%\right)=\frac{\mathrm{initial}\ \mathrm{drug}\ \mathrm{amount}-\mathrm{free}\ \mathrm{drug}\ \mathrm{amount}}{\mathrm{initial}\ \mathrm{lipid}\ \mathrm{amount}}\times 100 $$

The amount of doxycycline contained in NPs was investigated using HPLC equipped (SY-8100, China) with UV detector at a wavelength of 270 nm. The mobile phase was a mixture of ethanol (50%) and water (50%), the pump was of SY-8100 type, injector loop was at 772 PSI, and the column was of C-18 type (4.6 X 250 mm) is run at a flow rate of 1 mL/min. In this step, 10 mg of the sample alongside 10 ml of water and ethanol mixture were mixed and vortexed using sonication for 10 min to become homogenous. Then, it was centrifuged at 1500 rpm for 20 min and the supernatant was purified using 220 nm filters. A linear calibration curve with a good correlation coefficient (*r*^2^ = 0.9990) for concentrations in a range from 1 μg/ml to 250 μg/ml was achieved.

### Physical and chemical stability of NPs

The stability of NPs loaded with doxycycline was assessed in regular time intervals. After passing the NPs cross 450 nm filters, particle size, zeta potential and PDI were determined using nano Zetasizer device in the time intervals of 1 h, 24 h, 48 h, and also 1, 2, 4, 8, and 12 months after the preparation. At the same times, the concentration of doxycycline in NPs was determined using HPLC [14].

### In vitro release experiment

The lyophilized samples which were exactly weighted and enclosed in a dialysis bag (cut-off 12,000, Dialysis tubing, Sigma Chem. Co., Missouri, USA) were placed in a 40 ml release medium on the magnetic starring of 100 rpm at 37 °C. At predetermined time intervals, 0.5 ml of the medium was sampled and its doxycycline content was investigated using HPLC. For comparing the results of this set of experiments with those of free doxycycline, the same procedure was conducted in which free doxycycline were enclosed in the dialysis bags and placed in the same medium. Then at the same time intervals several samples were obtained from the medium and investigated. It should be noted that after each sampling from the medium, the new and fresh medium with the same amount was added [14].

### Fourier-transform infrared spectroscopy and differential scanning calorimetry

In the present study, a set of experiments was also conducted to discover any possible interaction between doxycycline and lipid compounds contained in DOX-SLN. FTIR analysis was performed to investigate such a possibility in a temperature range from 400 °C to 4000 °C [16].

DSC analysis was carried out to determine that the compound was at crystalline or amorphous form. The device was calibrated using indium for melting point and fusion heat. The heating rate was adjusted at 10 °C/min for the range of 20 to 400 °C. About 5 mg of DOX-SLN samples which were freeze-dried were placed on an aluminum plate to be analyzed. Moreover, a blank plate was used as the reference for each sample. For each sample, the experiment was repeated three times [17].

### Bacterial strains and cell line

For conducting in vitro experiments, the *B. melitensis M16* (obtained from Razi Vaccine and Serum Research Institute, Iran) was used. For the culture of the bacteria, brucella agar, tryptic soy broth (TSB) and tryptic soy agar (TSA) in the presence of 5% CO_2_ at 37 °C was used. The cell line of J774A.1(obtained from Pasteur Institute, Iran) was employed for the cell culture study. Moreover, for the culture of cell, Dulbecco’s Modified Eagle’s media (DMEM) medium with 10% FBS and 1% penicillin and streptomycin (pen-strep) was utilized in the presence of 5% CO_2_ at 37 °C [18, 19].

### Antibiotic sensitivity test under in vitro condition

Well diffusion and minimum inhibitory concentrations (MIC) tests were carried out in accordance with the guidelines of CLSI [20]. Each well was filled in by 100 μl of DOX-SLN and free doxycycline with various concentrations (50–25 and 12.5 μg/ml) and incubated at 37 °C in the presence of 5% CO_2_ for 24 h, 48 h and 72 h. After these time intervals, inhibition zone diameter was measured for each well and used as a basis for analyzing the antibacterial performance of DOX-SLN.

For performing the MIC test, sterilized 96-well cell culture plates were employed. All samples were sonicated to be homogenized before the test. The first concentration of NPs with the lowest antimicrobial concentration in the well diffusion test was selected. A series of concentrations was provided from them and 100 μl of each concentration was added to the wells of the micro plates. Next, 100 μl of Muller Hinton broth was added to all wells. Moreover, 5 μl of the bacteria solution was also added to all wells except the control one. The microplates were incubated at 37 °C in the presence of 5% CO_2_ for 24 h, 48 h, and 72 h. MIC was determined by visual examination of the turbidity and culturing the contents of the wells. The well with no bacterial growth was regarded as the minimum inhibitory concentration [20].

### In vitro cytotoxicity

MTT (3-(4,5-Dimethylthiazol-2-yl)-2,5-diphenyltetrazolium bromide) Assay test was performed using the MTT assay kit (Kiazist, Iran). The contents of the kit were as follows; MTT reagent, solubilizer, 2 Chanel reservoir, and 96-Well clean plate. In this step, 1 × 10^4^ J774A.1 were counted by trypan blue staining method and added to DMEM medium culture contained 10% FBS (fetal bovine serum) and 1% penicillin–streptomycin and placed in 96-well cell culture plates. After 24 h of incubation, the DMEM culture medium was removed and DOX-SLN, free doxycycline, and free SLN (blank sample) at various concentrations (25, 50, 100, 200, 400, 800 μl) alongside DMEM contained 10% FBS were added and incubated for 24 h. Several positive control samples which contained no drug were also provided. All experiments were repeated three times. Next, all cell were washed by FBS to all drugs and remained polymers be removed and 150 μl of fresh DMEM free of Fetal Bovine Serum was added to each well. Then, 20 μl of MTT Assay reagent was added to each well except the well associated with negative control sample. All plates were incubated at 37 °C in the presence of 5% CO_2_ for 3–4 h. After this period of time, 100 μl solubleizer was added to each well and mixed well using Orbital Shaker for 15 min to formazan particles be totally saluted. A 96-well ELISA plate reader was used to measure the absorption at 570 nm. The viability of the cells was calculated based on the degree of absorption of the positive control group (100% alive) [21].

### In vitro infection assay

The efficiency of DOX-SLN in comparison with free doxycycline to kill intra-cellular brucella was assessed using J774A.1 cells. Macrophages were cultured in 24-cell plates since 24-36 h before the infection. After reaching 90% confluence in each well and increasing the number of cells up to 5000 per well, 5 × 10^5^*B. melitensis* (the 1:100 ratio of cell to bacteria) were added to each well and incubated for 1 h. After phagocytosis, the culture medium was removed and replaced by a fresh culture medium containing 50 μg/ml Gentamicin and incubated for 1 h for killing of extracellular bacteria. Next, the culture medium was removed again and replaced by fresh culture medium containing 10% FBS and then incubated. Twenty four hours after the infection, the cells were washed by DMEM culture medium twice. Later again, 100 μl of DMEM containing 10% FBS was added to each well and different dilutions (25, 50, and 100 μl) of DOX-SLN, free doxycycline, and free SLN (blank) were added to infected cells, and incubated for different time interval, including 24, 48, and 72 h, at 37 °C in the presence of 5% CO_2_. Subsequently, the culture medium was removed and cells were washed by FBS twice. For the determination of intracellular bacterial load, cells were lysed in 250 μL of 0.1%Triton X-100™, and after being diluted were cultured on the Brucella agar culture medium. Colony forming units (CFUs) were counted after 24, 48, and 72 h of incubation at 37° in the presence of CO_2_ [10, 22].

### Statistical analysis of data

The Analysis of Variance (ANOVA) test was used to investigate the difference between the treatments. The Dunnett test was another statistical analysis performed for comparing groups. The confidence level was regarded as 95% and *P* < 0.05 was considered statistically significant.

## Results

### Properties of the NPs

The mean diameter of NPs in all formulations was 405 nm, which is relatively large for therapeutic objectives in the present study. However, in the optimum formulation (DOX-SLN-1), the mean diameter and PDI of the NPs were 299 ± 34 nm and 0.29 ± 0.027, respectively. The means of zeta potential for all formulations was − 17.57 mV and for the DOX-SLN-1 was − 28.7 mV (Table 1).

### Drug loading and encapsulation efficiency

The amount of doxycycline loaded on SLN in different formulations was between 8.2 and 11.3%. The amount of encapsulation was in the range of 91.3 to 97.4%. For DOX-SLN-1, the amounts of loaded and encapsulated doxycycline were 11.2% ± 1.3 and 94.9% ± 3.2, respectively (Table 1).

### NPs stability

Particle size, PDI and zeta potential were assessed at 0, 1st, 2nd, 4th, 8th, and 12th month of synthesis (Table 2). The size of NPs was nearly constant until the 8th month and the size difference was not significant. After 12 months, the size of NPs showed a 10.7% increase in diameter (from 299 to 320 nm).

**Table 2:** Technological characteristics of the DOX-SLN formulation: average diameter, polydispersity index and zeta potential during stability study, (Means ± SD, *n* = 3)

Technological parameters	Time (months)					
	0	1	2	4	8	12
Average diameter nm (±SD)	299±34	301±34	300±34	305±34	311±34	320±34
Polydispersity index	0.290 ±0.027	0.292±0.012	0.295±0.031	0.299±0.024	0.316±0.017	0.401±0.045
Zeta potential (mV ± SD)	−28.7±3.2	−29.2±2.9	−24.1±3.6	−31.9±4.0	−21.9±1.8	−26.2±2.7

### FE-SEM microscopy

**Fig. 1:**
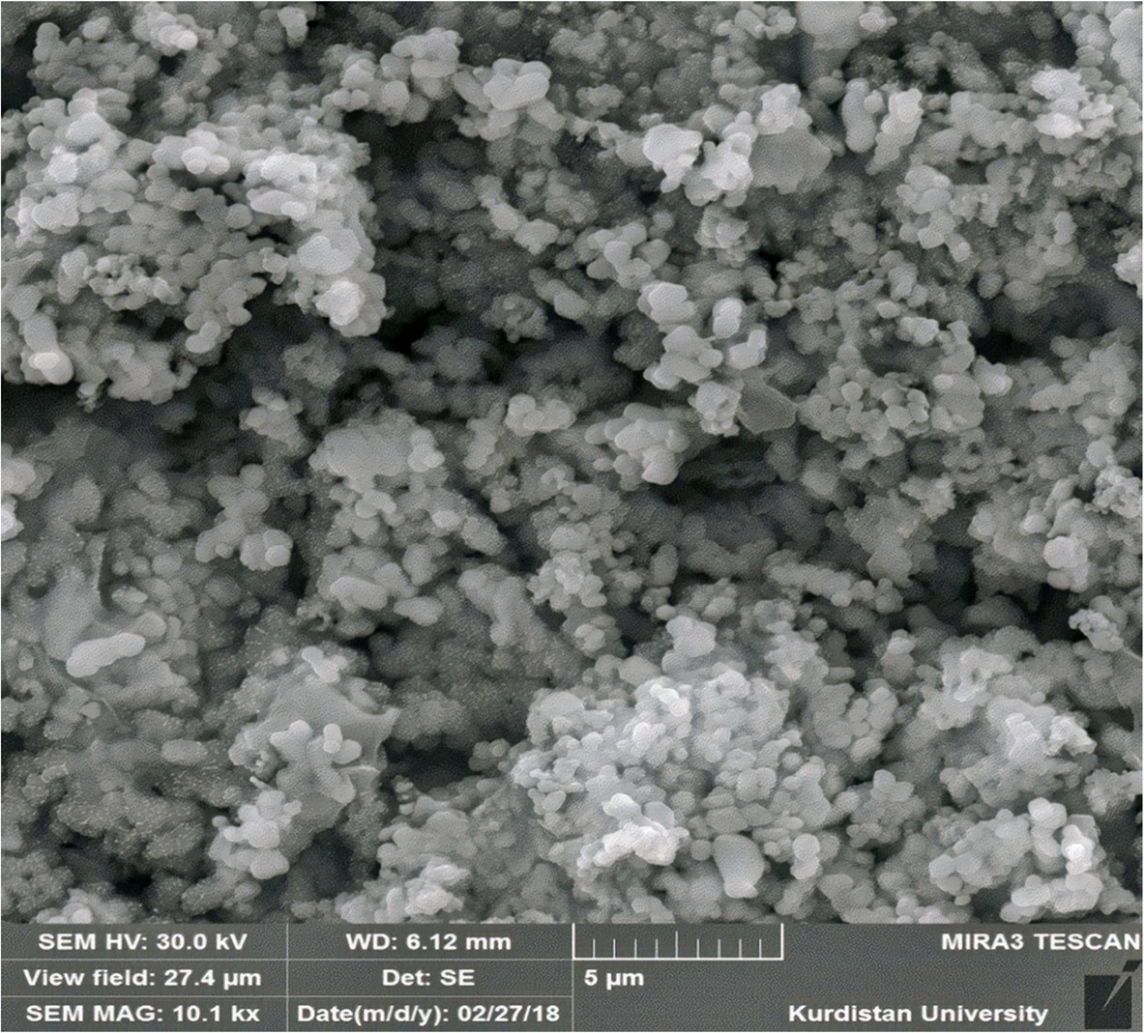
Field emission scanning electronic microscope images of DOX-SLN

The results of DOX-SLN-1 morphology analysis by FE-SEM is shown in Fig. 1. As seen, the majority of particles were spherical and had smooth surface with a homogeneous polydispersity. The mean diameter obtained by FE-SEM is significantly smaller than that obtained by PS, which is due to evaporating the SLN matrix during the SEM sample preparation at oven-drying process [23].

### FTIR analysis

**Fig. 2:**
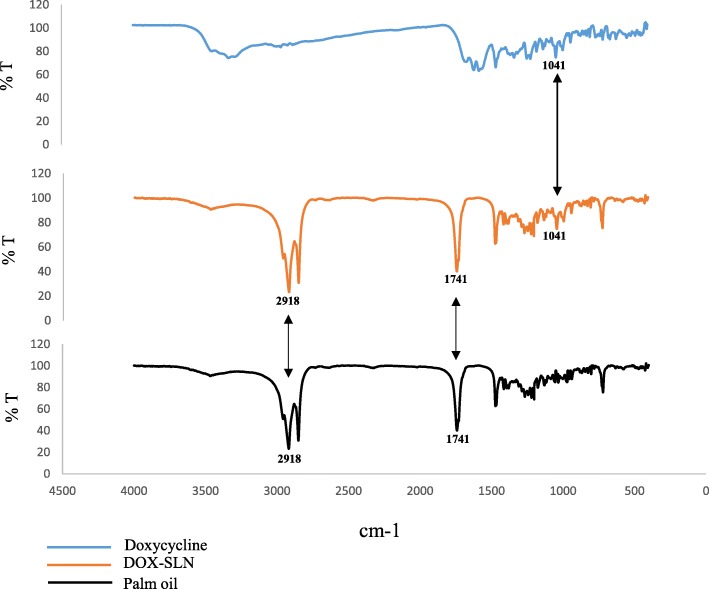
FTIR spectra of doxycycline, palm oil and DOX-SLN

The Fourier transform infra-red (FTIR) spectra of doxycycline, palm oil and SLN-DOX are presented in Fig. 2. As shown in Fig. 2, loading doxycycline into the SLN nanoparticles cause shielding the absorption peaks of drug at 400–1700 cm^− 1^, but three characteristic absorption peaks of doxycycline are still observed at 938, 993 and 1041 cm^− 1^ in the corresponding positions of SLN-DOX. Comparing the spectra of palm oil and SLN-DOX nanoparticles shows that main absorption peaks of palm oil at 1472, 1741 and 2918 cm^− 1^ appeared in the SLN-DOX nanoparticle spectra.

### DSC analysis

**Fig. 3:**
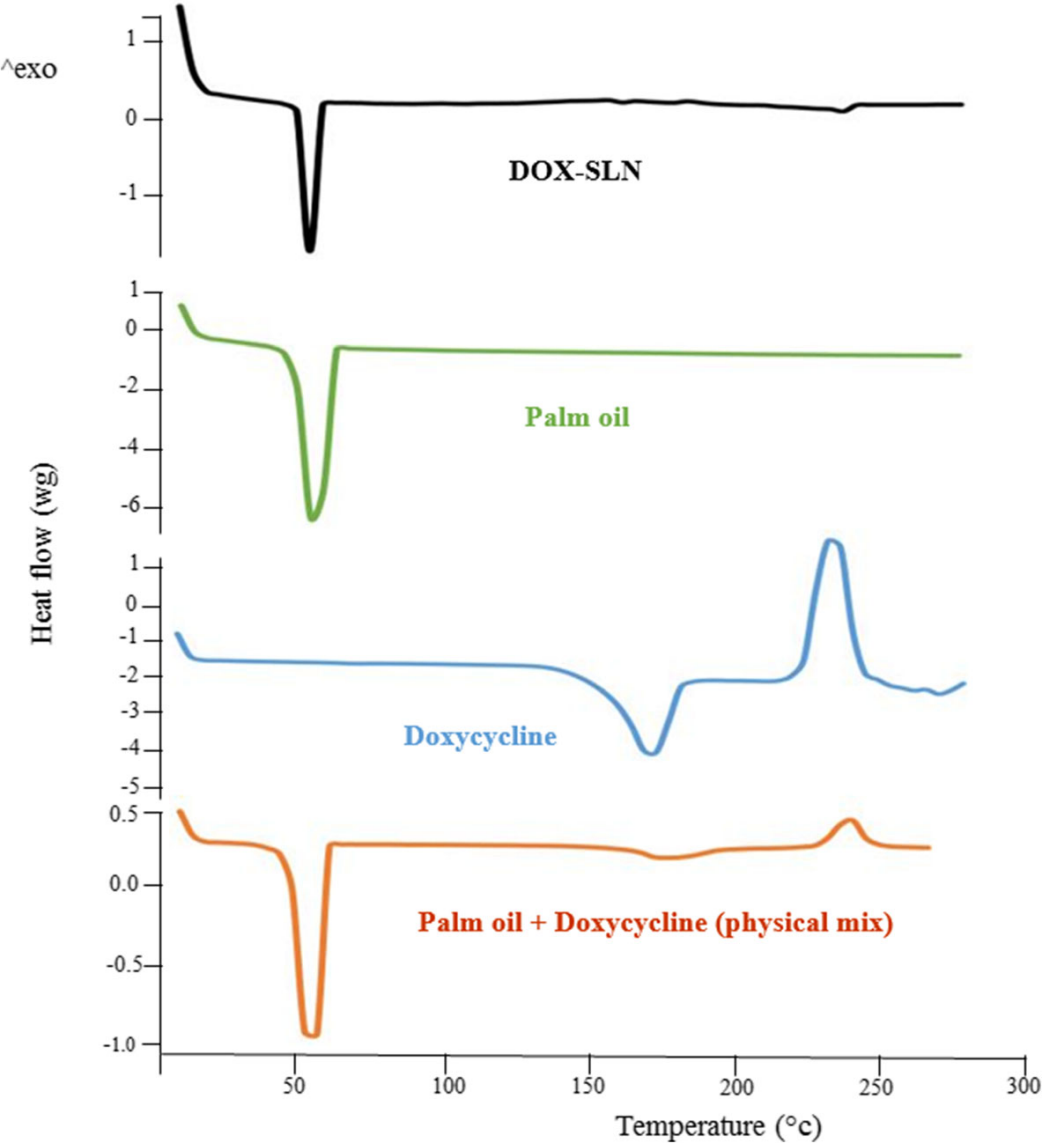
DSC thermograms of doxycycline, palm oil + doxycycline (physical mixture), palm oil and DOX-SLN

To investigate the recrystallization and melting behavior of SLN nanoparticles, DSC thermograms of DOX, palm oil, physical mixture and SLN-DOX nanoparticles are obtained (Fig. 3). DSC thermogram of palm oil shows a melting process at 63 °C. The melting points of physical mixture and SNL-DOX nanoparticles were similar to that of palm oil. However, a small change in the melting process of the pail oil in the physical mixture with the drug is observed which is in agreement with the previous studies [24]. In the DSC thermograms of DOX, a sharp endothermic peak was observed at 230 °C. Tracking this point for other compounds shows a small melting point for physical mixture and SLN-DOX. It is interesting that no any significant change was observed in the endothermic peak positions of DOX, physical mixture and SLN-DOX. The absence of a sharp melting peaks of DOX-SLN thermogram suggest that no free dox crystal remained in the SLN-DOX or DOX molecules are stabilized in the pail oil matrix [25].

### Drug release

**Fig. 4:**
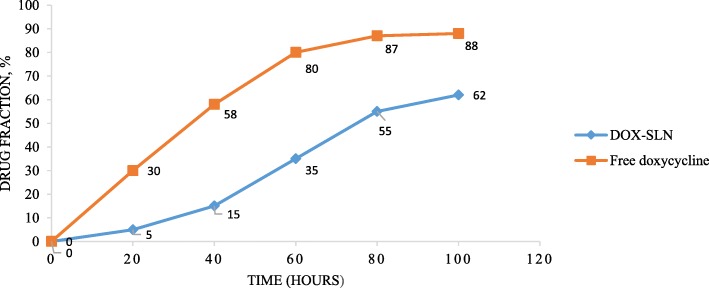
In vitro release profiles of doxycycline from the DOX-SLN formulation in pH = 7.4 phosphate buffer (*n* = 3). Free doxycycline was used as control

The profile of drug release (in vitro condition, pH = 7.4, PBS buffer) is shown in Fig. 4. The results demonstrated that the rate of free doxycycline release was higher than that of DOX-SLN-1, suggesting that DOX-SLN-1 is capable to control the release of encapsulated doxycycline (*P* = 0.04).

### Antibacterial studies

The results of well diffusion and MIC indicated in Table 3. doxycycline showed better antibacterial effects than DOX-SLN-1in well diffusion test and MIC. These observations were predictable because in both methods, the bacteria were in direct contact with the drug. After 72 h of incubation, the diameter of halo of growth inhibition was gradually increased, which indicates the slow release of the drug from DOX-SLN-1.

**Table 3:** Results of well diffusion and MIC test

Methods	Antibacterial activity																										
Well Diffusion	Zone of inhibition (mm) in three time (h) and three concentration(μg/ml)																										
	Doxycycline (50-25-12.5 μg/ml)									DOX-SLN (50-25-12.5 μg/ml)									Free SLN (blank) (50-25-12.5 μg/ml)								
	24 h			48 h			72 h			24 h			48 h			72 h			24 h			48 h			72 h		
	35	30	22	35	30	22	35	30	22	21	19	15	26	24	20	31	30	22	0	0	0	0	0	0	0	0	0
MIC	MIC value (μg/ml)																										
	doxycycline									Dox-SLN									free SLN (blank)								
	24 h									24 h			48 h			72 h			24 h			48 h			72 h		
	0.5									25			12.5			6.25			-			-			-		

### Toxic effects of NPs on cell

**Fig. 5:**
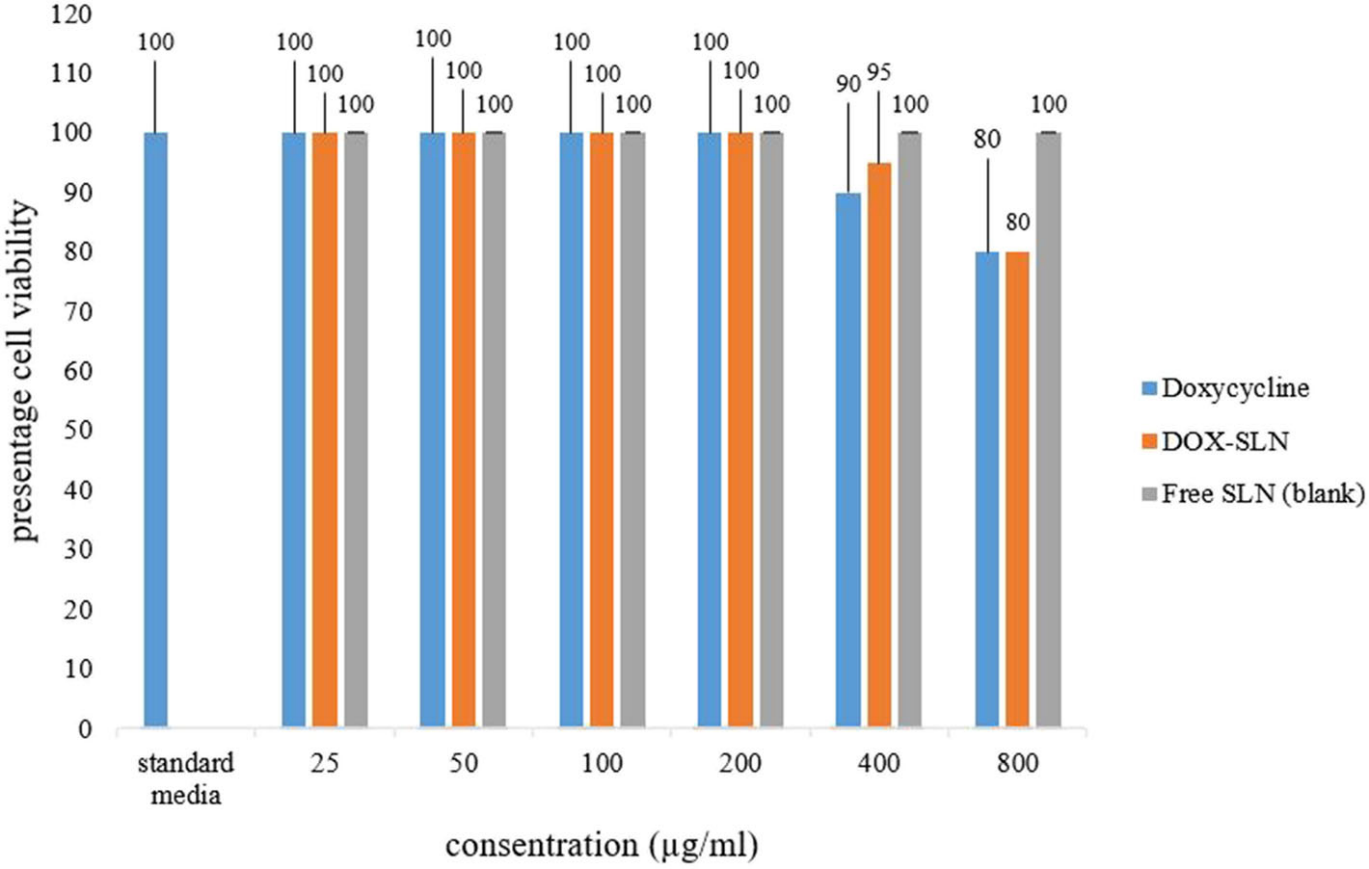
The effect of NPs on J774A.1 cells

The toxicity of different concentrations of DOX-SLN-1 and free SLN on cells is shown in Fig. 5. Cells with different concentrations of free doxycycline, DOX-SLN, and free SLN (blank) were incubated at 37 °C and the presence of 5% of CO_2_. Moreover, the same cells were incubated in the culture medium as the positive controls (without any treatment). All experiments were repeated three times. Cell viability was assessed using the MTT assay method. Absorption was measured at 570 nm. The basis of comparisons was 100% viability of positive controls.

The studied concentrations were much higher than the concentrations needed to treat infected macrophages under in vitro conditions.

### Intracellular infection study

The results of intracellular infection study showed that DOX-SLN-1 was able to reduce the number of colonies to as low as 3.5 log which was significantly lower than 5.4 log colonies obtained after the treatment by free doxycycline (*P* = 0.01). The number of colonies grown after treating with different concentrations of doxycycline, DOX-SLN, and free SLN are indicated in Table 4.

**Table 4:** In vitro efficacy of DOX-SLN compared to free doxycycline against *B. melitensis* inside J774A.1 cells

Treatment	Concentration (μg/ml)					
	100		50		25	
	Mean CFUs ± SEM	Log CFUs reduction	Mean CFUs ± SEM	Log CFUs reduction	Mean CFUs ± SEM	Log CFUs reduction
Doxycycline	5.4 ± 0.03	0.9	5.6 ± 0.01	0.7	6.1 ± 0.04	0.2
DOX-SLN	3.5 ± 0.07	2.8	3.9 ± 0.03	2.4	3.6 ± 0.01	2.7
Free SLN (blank)	6.3 ± 0.01	0	6.3 ± 0.01	0	6.3 ± 0.01	0
Negative Control	6.3 ± 0.02	0	6.3 ± 0.02	0	6.3 ± 0.02	0

## Discussion

Chronic infectious diseases such as brucellosis impose a considerable economic burden on societies. In addition, since the bacteria are inside the cell, they are protected from the body immune system but antibiotics that are presented in extracellular environment [21]. Therefore, it is necessary to develop drug delivery systems to achieve better treatment of intracellular infections. The aim of the present study was to investigate the effect of DOX-SLN on intra-Cellular *B. melitensis* in order to develop a more effective and consistent drug delivery system for bacteria inside the macrophages. The results indicated that DOX-SLN is significantly more effective in reducing the number of bacteria than free Doxycycline (*P* = 0.01), suggesting that the use of NPs resulted in the continuous and consistent presence of drug at the target site.

Brucella spp. are facultative intracellular pathogens that mainly infect and replicate inside liver and spleen cells or the mononuclear phagocytic system (MPS). After capturing by these cells, the organism presents an excellent model for escaping to remain safe from regular phagosome maturation process. After ingestion, most of them are in cell vacuoles retaining late endosomal/lysosomal markers (LAMP-1 positive) which will eventually be killed while a few of these vacuoles survive by avoiding lysosomal fusion. Brucella lipopolysaccharide surface and cyclic beta-1, 2-glucan (which are essential for bacterial survival and replication, from lipid rafts to phagosomes) has been suggested to play a role in the control of the phagosomal maturation. Anyway, those vacuoles that successfully evade lysosomal fusion are capable of interacting with ER exit sites. Afterward, they fuse to ER to generate ER-derived replicative Brucella-containing vacuoles which acidification become substantial to trafficking to the ER and subsequent intracellular replication of the bacteria [26].

The obtained diameter (299 ± 34 nm) for DOX-SLN is an appropriate size to be phagocytosed by phagocytes. The results of the present study demonstrated that as the sonication time increases, the size of nanoparticle decreases. The results are in line with those of Liu et al. [27]. The present study also demonstrated that the size of SLN increased after drug loading. In the other words, the size of DOX-SLN is larger than free SLN. Moreover, the size of nanoparticle increased after lyophilization, which is consistent with the findings of a study carried out by Chantaburanan et al. [16]. Severino et al. prepared SLN using high pressure homogenization method and obtain nanoparticle of 439.5 nm in size [28]. Chetoni et al. applied 80 nm NPs in eye drops [14].

The mean PDI of the NPs in our study were 0.29 ± 0.027. Since a PDI value lower than 0.3 has been reported as an ideal index, the size distribution of NPs was therefore satisfactory [15]. In the present study, it was revealed that as the time of homogenization using ultrasonic increased, the size of NPs tended to smaller and more homogenous, which is in line with those of previous studies, such as Ding et al. and Marquele-Oliveira et al. [29, 30].

The mean value of zeta potential for the DOX-SLN was − 28.7, which is good enough to prevent NPs to be agglomerated and formed large colloids. This resulted in physical stability of the nanoparticle. Increasing the amount surfactant resulted in increase of zeta potential. As the double emulsion method is a 2-step method, so two types of surfactant were used, accordingly the zeta number obtained in the present study is rationale [13, 27].

Nowadays, many different NPs are used in order to drug delivery. The levels of drug loading and encapsulating for different NPs depends on materials used for their synthesis and methods of preparation [31]. The drug loading and encapsulating efficiency in the present study were 11.2% ± 1.3 and 94.9% ± 3.2, respectively; which was similar to those of other studies. D Liu et al. have produced a nanoparticle using modified emulsion/solvent evaporation method and phospholipids for loading the hydrophilic diclofenac sodium. Similar to the present study, they applied two methods of spectrophotometer and HPLC for assessing the rate of drug loading and encapsulation. They reported the rate of drug loading and encapsulation as 6.5 and 72.9%, respectively, which is similar to the results of the present study [27]. Other factors able to affect encapsulation efficiency is the amount of surfactant used during the preparation of NPs [14, 32].

The double emulsion/melt dispersion technique is more effective in loading and encapsulation of doxycycline in NPs than other techniques such as the modified solvent removal method, the hot homogenization and ultrasonication method, and the high shear homogenization-ultrasonication method [33–35].

In the current study, after 12 months, the size of NPs showed a 10.7% increase in diameter (from 299 to 320 nm). This can be because of agglomeration of NPs. However, no significant change was observed for PDI and zeta potential. These levels of alteration can be acceptable because the NPs were used to assess their effects on the macrophages in experimental conditions.

In the study carried out by Dong et al., the stability of NPs was investigated in a 6-month time period and it was concluded that the NPs size and PDI became larger, while no change was observed in the zeta potential and loading rate [27]. Chetoni et al. also loaded tobramycin in SLN and observed no change in nanoparticle characteristics for a time period of 12 months, while after 24 months, the size of NPs increased from 80 nm to 120 nm [14].

The release time of doxycycline from DOX-SLN in the current study was almost 72 h. However, other studies have reported different release times which is mainly due to the use of different methods for producing NPs [14, 15, 29].

Most studies have outlined that the release time of drug from nanoparticle is higher than those of their controls, which is due to the slow release of drug from lipid matrix. Moreover, previous studies have demonstrated that as the size of nanoparticle decreases the release time also decreases, which is due to the increase in the surface area of smaller particles [17, 36].

Imbuluzqueta et al. utilized the gentamycin loaded in PLGA for killing *B. melitensis* phagocytosized by macrophages, which had a lower efficacy than DOX-SLN synthesized in the present study [26]. Another study conducted by Seleem et al. used doxycycline loaded in Nanoplexes for killing *B. melitensis* in J774A.1 cells. They observed no significant difference between the efficacies of the drug loaded in NPs and the corresponding free drug [22]. The results are in contradiction with those of the present study, which can be due to the rapid release of the drug from the NPs; the release time reported by that study was 15 h which is significantly lower than 72 h we observed in the current study.

There were a number of limitations to our study including: studying on *B. melitensis* was very difficult due to its pathogenic nature. Because we had many tests and errors to set up an appropriate nanoparticle, the duration of the project was long. This project needs many equipment, as all of them were not centralized in one place, the work became very hard.

## Conclusions

The double emulsion method is suitable to encapsulation of DOX as a hydrophilic drug so that particle size, zeta potential, and PDI were desirable for our objectives. The time required for the complete release of drug and therefore its antibacterial effectiveness was 72 h. The effects of DOX-SLN on the bacteria enclosed in macrophages was significantly higher than the free DOX. This can be attributed to the phagocytosis of NPs by macrophages and slow continuous release of drug. The application of these NPs can be considered as a promising tool for treating intracellular bacteria, particularly *B. melitensis* and preventing the relapse of the disease.

Suggestions that can help researchers interested in this topic: using multiple-drug combination that are routinely used to treat brucellosis, investigating nanoparticle’s effect and toxicity in in vivo conditions, utilizing other nanoparticles and comparing its effect on *B. melitensis* with SLN.

## Abbreviations

DMEM: Dulbecco’s Modified Eagle’s media
DOX-SLN: Doxycycline-loaded Solid Lipid Nanoparticles
DSC: Differential scanning calorimetry
FBS: Fetal bovine serum
FE-SEM: Field emission scanning electronic microscope
FTIR: Fourier-transform infrared spectroscopy
HPLC: High-performance liquid chromatography
MIC: Minimum Inhibitory Concentration
MTT: 3-(4,5-Dimethylthiazol-2-yl)-2,5-diphenyltetrazolium bromide
NPs: Nanoparticles
PDI: Polydispersity index
SLN: Solid Lipid Nanoparticles
TSA: Tryptic soy agar
TSB: Tryptic soy broth
WHO: World Health Organization
